# The Tocotrienol-Rich Fraction Is Superior to Tocopherol in Promoting Myogenic Differentiation in the Prevention of Replicative Senescence of Myoblasts

**DOI:** 10.1371/journal.pone.0149265

**Published:** 2016-02-17

**Authors:** Shy Cian Khor, Azraul Mumtazah Razak, Wan Zurinah Wan Ngah, Yasmin Anum Mohd Yusof, Norwahidah Abdul Karim, Suzana Makpol

**Affiliations:** Department of Biochemistry, Faculty of Medicine, Level 17, Preclinical Building, Universiti Kebangsaan Malaysia Medical Centre, Jalan Yaacob Latif, Bandar Tun Razak, 56000 Cheras, Kuala Lumpur, Malaysia; University of Minnesota, UNITED STATES

## Abstract

Aging results in a loss of muscle mass and strength. Myoblasts play an important role in maintaining muscle mass through regenerative processes, which are impaired during aging. Vitamin E potentially ameliorates age-related phenotypes. Hence, this study aimed to determine the effects of the tocotrienol-rich fraction (TRF) and α-tocopherol (ATF) in protecting myoblasts from replicative senescence and promoting myogenic differentiation. Primary human myoblasts were cultured into young and senescent stages and were then treated with TRF or ATF for 24 h, followed by an analysis of cell proliferation, senescence biomarkers, cellular morphology and differentiation. Our data showed that replicative senescence impaired the normal regenerative processes of myoblasts, resulting in changes in cellular morphology, cell proliferation, senescence-associated β-galactosidase (SA-β-gal) expression, myogenic differentiation and myogenic regulatory factors (MRFs) expression. Treatment with both TRF and ATF was beneficial to senescent myoblasts in reclaiming the morphology of young cells, improved cell viability and decreased SA-β-gal expression. However, only TRF treatment increased BrdU incorporation in senescent myoblasts, as well as promoted myogenic differentiation through the modulation of MRFs at the mRNA and protein levels. *MYOD1* and *MYOG* gene expression and myogenin protein expression were modulated in the early phases of myogenic differentiation. In conclusion, the tocotrienol-rich fraction is superior to α-tocopherol in ameliorating replicative senescence-related aberration and promoting differentiation via modulation of MRFs expression, indicating vitamin E potential in modulating replicative senescence of myoblasts.

## Introduction

Sarcopenia is a geriatric syndrome that is characterized by a dramatic loss of skeletal muscle mass and strength in advancing age. Although the underlying mechanism of these alterations is not clear, several risk factors have been considered, such as immobilization, chronic diseases, hormone and pro-inflammatory cytokine shift and malnutrition in the elderly [1]. Loss of muscle regenerative capacity has been suggested as one of the possible contributory factors of this age-related muscle deterioration [2].

Skeletal muscle has an established regeneration competency in restoring and maintaining muscle mass when muscle cells undergo injury [3]. Muscle regeneration essentially involves four sequential and overlapping phases: degeneration, inflammation, regeneration and remodeling. Satellite cells are the key regenerative phase and will be activated, proliferate and differentiate in response to stimuli. Proliferating satellite cells are known as myoblasts [4]. In addition to producing functional progeny via differentiation, satellite cells can replicate to maintain the satellite cell pool; thus, they are also categorized as muscle stem cells [5]. The heterogeneity of satellite cells has provoked the rationale of targeting these cells for therapeutic purposes in ameliorating age-related sarcopenia or pathological dystrophic muscle [6].

In aging, satellite cells malfunction and fail to sustain their normal quiescent state, irrevocably influencing their regenerative and self-renewal capacities [7]. A decreased number of satellite cells in old age were also observed [6,8]. However, this decrease may not be the sole reason for the gradual loss of muscle rejuvenation capacity in old age. In fact, a permissive atmosphere is imperative rather than the number of satellite cells, whereby satellite cells from old muscle can be engaged for myogenic activity when exposed to a young systemic environment [9–12].

Myogenic differentiation is regulated by a family of myogenic regulatory factors (MRFs) that includes MyoD, Myf5, Myogenin and MRF4. MRFs are transcription factors with a basic helix–loop–helix (bHLH) central domain that assist protein interactions and DNA binding to activate muscle-specific genes [4]. The deregulation of Myf5, MyoD and myogenin at an early stage of differentiation is interrelated with the differentiation capability of senescent myoblasts, resulting in the formation of smaller myotubes that resemble the condition in sarcopenia [13,14]. Thus, ongoing research in finding ways to restore the regenerative capacity in old myoblasts will presumably provide precious insight for combating muscle atrophy in aging or degenerative diseases.

Because muscle atrophy or aging itself is closely related to oxidative stress, the re-establishment of redox balance should be potentially advantageous in the amelioration of age-related muscle wasting [15,16]. Vitamin E is a lipid-soluble vitamin that is able to scavenge free radicals, boosts cellular antioxidant competency and prevents oxidative damage. There are two subgroups of vitamin E: tocopherols and tocotrienols [17]. Howard et al. reported that α-tocopherol (ATF) was able to repair the laser-induced disrupted membrane of myoblasts, which supports a therapeutic effect exerted by vitamin E [18]. A significant correlation between the ATF level and sarcopenia indicators among the elderly has been reported [19]. Vitamin E deficiency will not only affect muscle performance but also accelerate the progression of aging [20]. Therefore, it is rational to introduce antioxidants, such as vitamin E, to prevent sarcopenia, even though further studies are still required [16].

Human myoblasts can be isolated and cultured *in vitro* with a limited proliferation capacity, whereby at a certain stage, they will undergo growth arrest, termed replicative senescence [21]. The present study was designed to elucidate the effects of the tocotrienol-rich fraction (TRF) and α-tocopherol (ATF) in ameliorating senescent myoblasts and promoting myogenic differentiation during replicative senescence.

## Methods

### Cell Culture and Replicative Senescence Model

Primary human myoblasts (Human Skeletal Muscle Myoblasts; HSMM) at passage 2 from two donors, a 17-year-old Caucasian female and a 16-year-old Caucasian male were purchased from Lonza (Walkersville, MD, USA). Briefly, myoblasts were cultured in Skeletal Muscle Basal Medium (SkBM) that was supplemented with human epidermal growth factor, fetal bovine serum, dexamethasone, L-glutamine, and gentamicin sulfate/amphotericin B (Lonza, Walkersville, MD USA). Cells were cultivated at 37°C in a humid atmosphere containing 5% CO_2_. The myoblasts then underwent serial passaging to reach senescence. For each passage, the population doublings (PD) of cells was calculated as: In (*N*/*n*)/In2, where *N* is the number of cells at harvest stage, and *n* is the number of cells at seeding stage [14]. The starting PD in this study was 8. The cells achieved replicative senescence when they were unable to proliferate within 10 days in culture, even with consecutive replenishment.

### Analysis of Cell Morphology and Myogenic Purity

Myogenic purity and myoblasts morphology were observed by the immunocytochemistry method using a mouse monoclonal anti-Desmin antibody (D33; Dako, Produktionsvej, Denmark). Myoblasts were plated in μ-Slide 8 well (ibidi, Martinsried, Germany) at a density of 1×10^4^ cells per well. The cells were fixed in cold ethanol. Then, anti-Desmin antibody (1:50) and Alexa Fluor 488 goat anti-mouse (Life Technologies, Carlsbad, CA, USA) were used to incubate the myoblasts in sequence. Nuclei were visualized using Hoechst 33342 (Life Technologies, Carlsbad, USA). The slides were then viewed under a Confocal Laser Scanning Microscope Leica TCS SP5 II, and data were acquired using LAS AF version Lite 2.6 software (Leica Microsystems, Wetzlar, Germany). To determine the percentage of desmin-positive cells, a minimum of 50 cells were counted in three independent cultures. In addition, the morphological changes of myoblasts were observed, while the width and length of myoblasts were visualized and measured using LAS AF version Lite 2.6 software (Leica Microsystems, Wetzlar, Germany). For each group of cells, at least 30 cells were analyzed.

### Determination of DNA Synthesis in Proliferating Cells

The amount of 5-bromo-2’-deoxyuridine (BrdU) incorporation indicates the total proliferating cells. Thus, the cell proliferation ELISA, BrdU (colorimetric) kit (Roche, Penzberg, Germany) was used to determine the effects of replicative senescence, TRF and ATF on cell proliferation. This immunoassay was performed according to the manufacturer’s instructions. The cells were labeled with BrdU, a pyrimidine analog that will incorporate into the DNA and was detected by a microtiter plate reader (VersaMax Molecular Devices, USA) at 450 nm with reference to 690 nm.

### Determination of Senescence Biomarkers

The expression of SA-β-gal was determined as described by Dimri et al. [22] in order to confirm the presence of senescent myoblasts. This process was carried out using a Senescent Cell Histochemical Staining Kit (Sigma-Aldrich, St. Louis, Missouri, USA) according to the manufacturer’s instructions. Cells were incubated in staining solution for 8 hours at 37°C in the absence of CO_2_ before analysis. At least 100 cells were observed, and the percentage of blue stained cells was calculated. In addition, the morphological changes of myoblasts were also observed.

### Preparation of Vitamin E Treatments

TRF Gold Tri E 70 (Sime Darby Sdn. Bhd., Selangor, Malaysia) and ATF (Malaysian Palm Oil Board, Selangor, Malaysia) were used as treatments in this study. Briefly, stock solutions of TRF were freshly prepared in 100% ethanol (1:1) and kept at −20°C for no more than one month. A similar process was applied for ATF preparation. TRF and ATF were then incubated overnight with fetal bovine serum at 37°C before use. The cell viability was assessed with a CellTiter 96^®^ Aqueous Non-Radioactive Cell Proliferation Assay (MTS; Promega, Madison, WI USA) according to the manufacturer’s instruction. Various concentrations of TRF or ATF were used to treat the cells for 24 hours. Then, MTS was added and further incubated for 2 hours. The absorbance of MTS formazan was measured at 490 nm with a microtiter plate reader (VersaMax Molecular Devices, USA). The optimum dose of treatments was used for subsequent experiments.

### Induction of Myogenic Differentiation

To induce muscle cell differentiation, the proliferating medium SkBM was replaced with DMEM:F12 (Lonza, Walkersville, MD USA) that was supplemented with 2% horse serum (ATCC, Baltimore, USA). The differentiation medium was changed every 2 days until the desired day of differentiation for parameter measurement.

### Analysis of Myogenic Differentiation

To evaluate the efficiency of differentiation, a micro-insert 4 well, μ-Dish was used (ibidi, Martinsried, Germany) to culture the cells to determine myotubes formation. After 9 days of differentiation, myotubes were stained using an anti-Desmin antibody. The fusion index and the size of myotubes were calculated, indicating myotube formation. The formula below was used to calculate the fusion index, and a minimum of 50 nuclei were counted in 3 different randomly chosen optical fields.

$$\text{Fusion Index}=\frac{\text{The number of nuclei in myotubes }(>\;2\;nuclei)}{\text{The total number of nuclei in desmin}-\text{positive cells}}\times 100\text{\%}$$

To determine the size of the myotubes, the number of nuclei per myotube was counted in a minimum of 11 multinucleated cells in 3 different randomly chosen optical fields.

### Determination of MRFs at an Early Phase of Myogenic Differentiation

At days 0, 1 and 2 of differentiation, total RNA was extracted using the TRI reagent (Molecular Research Center Inc., Ohio, USA). For gene expression determination, quantitative real-time RT-PCR (qRT-PCR) was used. The expression of *MYF5*, *MYOD1* and *MYOG* mRNA was quantitatively analyzed using a one-step qRT-PCR technique. qRT-PCR was performed with 100 ng of total RNA, 400 nM each primer and KAPA SYBR FAST One-Step qPCR kit (Kapa Biosystems, Boston, Massachusetts, USA) according to the manufacturer’s instructions. The primer sequences are *GAPDH* forward 5’-TCCCTGAGCTGAACGGGAAG-3’, *GAPDH* reverse 5’-GGAGGAGTGGGTGTCGCTGT-3’, *MYF5* forward 5’-TCACCTCCTCAGAGCAACCT-3’, *MYF5* reverse 5’-ATTAGGCCCTCCTGGAAGAA-3’, *MYOD1* forward 5’-CGCCAGGATATGGAGCTACT-3’, *MYOD1* reverse 5’-GAGTGCTCTTCGGGTTTCAG-3’, *MYOG* forward 5’-CAGTGCCATCCAGTACATCG-3’ and *MYOG* reverse 5’-AGGTTGTGGGCATCTGTAGG-3’. The master mix was prepared, and PCR reactions were carried out in a Bio-Rad iQ5 Cycler (Hercules, CA, USA) with the following programmed reaction profile: cDNA synthesis for 5 min at 42°C; pre-denaturation for 4 min at 95°C; and PCR amplification for 40 cycles of 3 sec at 95°C and 20 sec at 60°C. These reactions were followed by a melt curve analysis to determine the reaction specificity and the expression of each targeted gene. The expression level of each targeted gene was normalized to that of glyceraldehyde 3-phosphate dehydrogenase (*GAPDH*). The relative expression value (REV) was calculated using the 2^-ΔΔCt^ method of relative quantification and the following equation: REV = 2 ^Ct value of GAPDH -Ct value of the gene of interest^. Then, the fold change of expression was determined.

### Determination of Myogenin Expression

At day 3 of differentiation, the number of cells expressing myogenin was estimated using a mouse monoclonal anti-myogenin antibody (F5D, Dako, Produktionsvej, Denmark) at a 1:20 dilution overnight at 4°C. Alexa Fluor 488 was used as the secondary antibody. Nuclei were visualized using Hoechst 33342. The cells were observed under an EVOS FL Digital Inverted Fluorescence Microscope (Life Technologies, Carlsbad, USA).

### Assessment of Intracellular Free Radical Generation

In order to measure free radicals generation by myoblasts, we used two types of dyes, i.e. dihydroethidium (DHE) and 5-(and-6)-carboxy-2′,7′-dichlorodihydrofluorescein diacetate (carboxy-H_2_DCFDA) (Molecular Probes, Eugene, OR, USA). The DHE-stained cells indicated oxidation by superoxide anion, while carboxy-H_2_DCFDA is oxidized by hydrogen peroxide (H_2_O_2_), peroxynitrite or hydroxyl radical. Superoxide anions may contribute to carboxy-H_2_DCFDA oxidation albeit at a lesser degree. Briefly, myoblasts were incubated in 20 μM of DHE and 40 μM of carboxy-H_2_DCFDA for 45 min. After that, the cells were washed with PBS and recovered in medium for 30 minutes. Then, we measured the intensity by using microplate reader (Infinite^®^ 200, Tecan, USA) at excitation/emission wavelength (Ex/Em) 518/600 nm and 488/521 nm respectively.

### Statistical Analysis

Statistical analyses were performed using SPSS 17.0 software (IBM, NY, USA). All of the data are reported as the means ± standard deviation (SD) from at least three replicates. For all of the tests, p<0.05 was considered statistically significant. To determine significance between two treatment groups, comparisons were made using an independent T-test, while ANOVA was used to analyze multiple groups, followed by a *post-hoc* Tukey HSD or LSD (if equal variance was assumed) and Dunnett T3 (if equal variance was not assumed) tests.

## Results

### Replicative Senescence Model of Myoblasts: Characteristics and Proliferation

**Fig 1:**
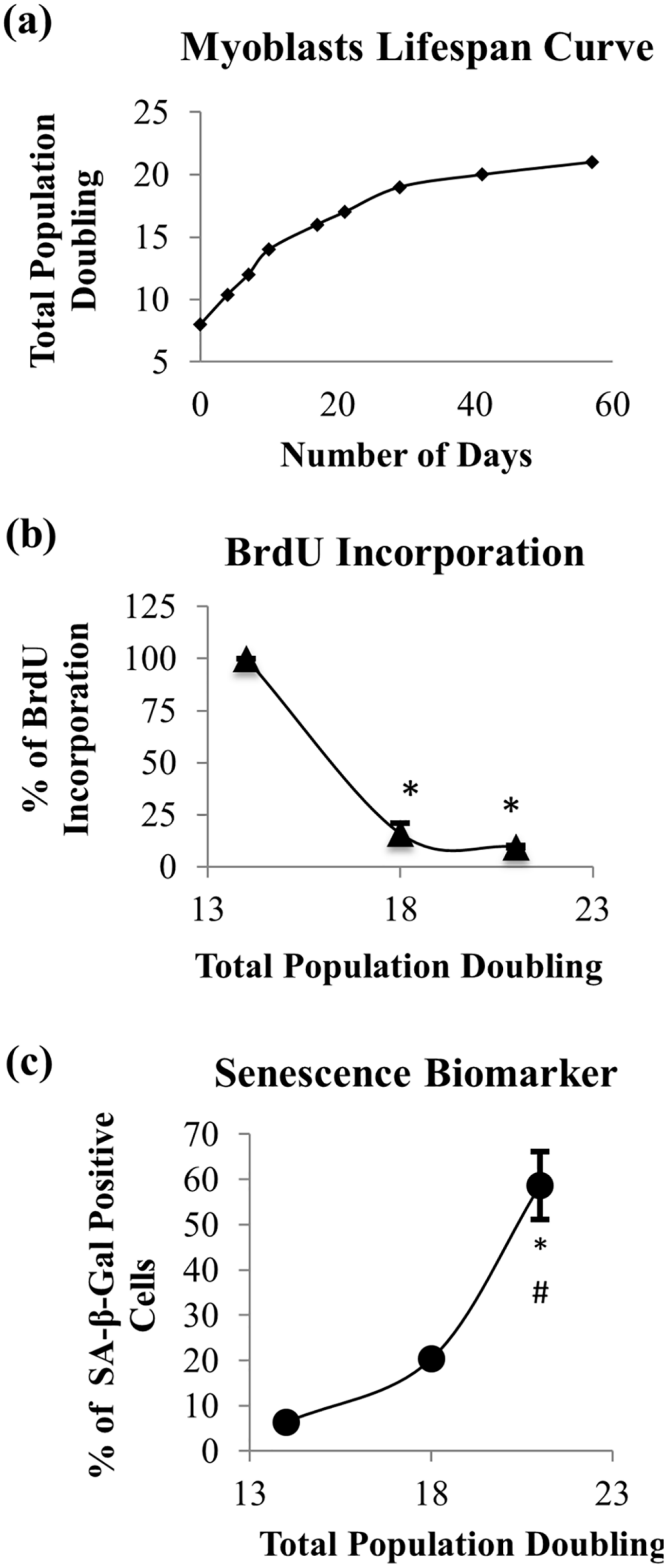
Effects of serial passaging on population doubling, cell proliferation and expression of SA-β-gal in myoblasts. During extensive expansion, myoblasts significantly lost their proliferation capacity as represented by a hyperbolic proliferative lifespan curve (a) and decreasing percentage of BrdU incorporation (b), while the percentage of senescent cells increased, as represented by positive SA-β-gal staining (c). For (b) and (c), the data are presented as the means ± SD, n = 3. *p<0.05 compared to myoblasts at PD14 (young), ^#^p<0.05 compared to myoblasts at PD18 (pre-senescent) with a *post-hoc* Dunnett T3.

To elucidate the effects of aging on myoblasts, we expanded the cells until replicative senescence. The lifespan curve that was plotted based on their cumulative PD showed that myoblasts have a limited proliferative capacity, and cell growth was halted at 21 divisions in culture (Fig 1a). Based on BrdU incorporation, the proliferation of myoblasts decreased with increasing total PD, whereby the percentage of BrdU incorporation at PD18 and PD21 was significantly different compared to the percentage of BrdU incorporation at PD14 (p<0.05) (Fig 1b). The percentage of SA-β-gal-stained cells increased with the serial passaging of myoblasts, which was significantly higher at PD21 compared to both PD14 and PD18 (p<0.05) (Fig 1c). Therefore, myoblasts are considered young at PD<15 and senescent at PD>20. No loss of myogenicity was observed during the replicative senescence of myoblasts, as indicated by the presence of desmin in 96% of the cell population (Table 1), allowing a reliable statistical comparison of these cells.

**Table 1:** Percentage of desmin-positive cells in different cell stages.

Myoblasts	Young(PD14)	Presenescent(PD18)	Senescent(PD21)
**Desmin +ve**	96.67±2.31(n = 3)	98.67±1.15(n = 3)	96.00±2.00(n = 3)

### Promotion of Cell Viability and Proliferation

**Fig 2:**
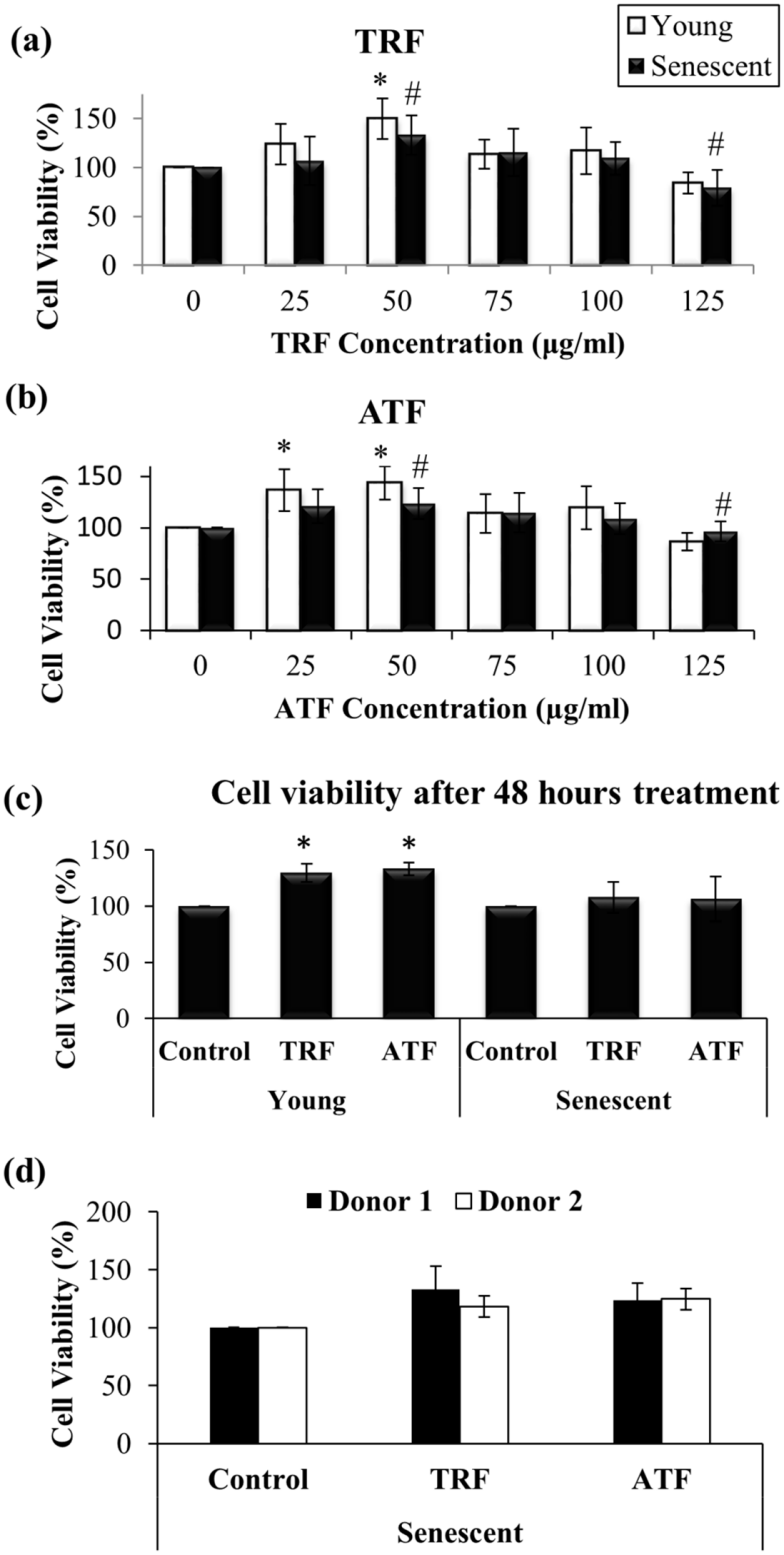
Effects of the TRF and ATF treatments on cell viability and proliferation. Dose-response curve of TRF (a) and ATF (b) treatments (24 h) in young and senescent myoblasts (n = 9). The prolonged treatments of TRF and ATF (48 h) at optimal dose were unlikely to further improve cell viability in senescent myoblasts (c). Therefore, 24 h treatment was used in subsequent experiment. Comparison of cell viability between myoblasts from donor 1, a 17 year-old, female Caucasian and donor 2, a 16 year-old male Caucasian (d). There were no significant different observed between the two cell lines in response to both TRF and ATF treatments based on the viability assessment. *Post-hoc* Dunnett T3 test for (a) and (b). *p<0.05 significantly different compared to untreated young myoblasts, ^#^p<0.05 significantly different compared to untreated senescent myoblasts, ^§^p<0.05 significantly different compared to TRF-treated senescent myoblasts. The data are presented as the means ± SD.

Incubation with various concentrations of TRF or ATF for 24 h significantly increased the viability of young and senescent myoblasts (Fig 2a and 2b). Cells that were treated with TRF and ATF at concentrations of 50 μg/ml, exhibited the greatest percentage of viability in both myoblasts. Therefore, in the subsequent experiments, 50 μg/ml TRF and 50 μg/ml ATF were used for treatment in young and senescent myoblasts. Prolonged treatment (48 hours) using the optimal dose (50 μg/ml) improved cell viability in young cells, but not in senescent myoblasts (Fig 2c). Subsequently, the optimal dose was applied on senescent myoblasts from the other donor (a 16-year-old Caucasian male). However, there was no significant different observed on the cell viability as compared to the viability of myoblast from the first donor (a 17-year-old Caucasian female) (Fig 2d). Therefore, for the following experiment, myoblasts from the first donor was used.

**Fig 3:**
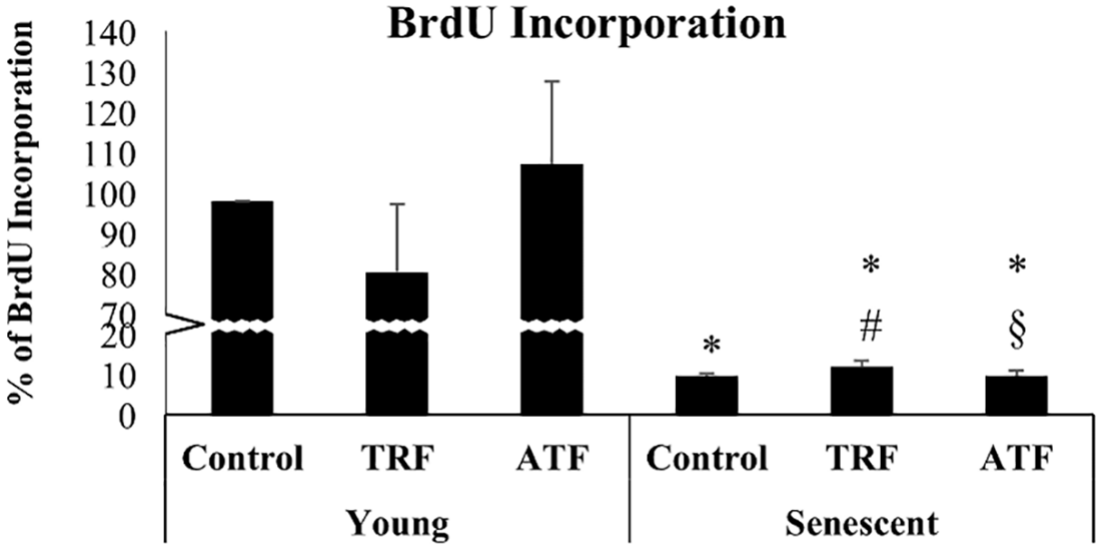
Effects of TRF and ATF on BrdU incorporation. Cells were treated with optimal dose of TRF and ATF followed by cell proliferation determination based on the percentage of BrdU incorporation (n = 3). Only TRF-treated senescent myoblasts showed increased BrdU incorporation indicating promotion of cell proliferation and DNA synthesis with TRF treatment. *p<0.05 significantly different compared to untreated young myoblasts, #p<0.05 significantly different compared to untreated senescent myoblasts, §p<0.05 significantly different compared to TRF-treated senescent myoblasts, with *post-hoc* LSD test. The data are presented as the means ± SD.

A significantly decreased percentage of BrdU incorporation was observed in senescent myoblasts compared to young cells (p<0.05). Treatment with TRF increased the percentage of BrdU incorporation in senescent myoblasts (p<0.05) (Fig 3), while no significant difference was observed in young myoblasts that were treated with TRF or ATF.

### Improvement in Myoblasts Cellular Morphology with TRF and ATF Treatment

**Fig 4:**
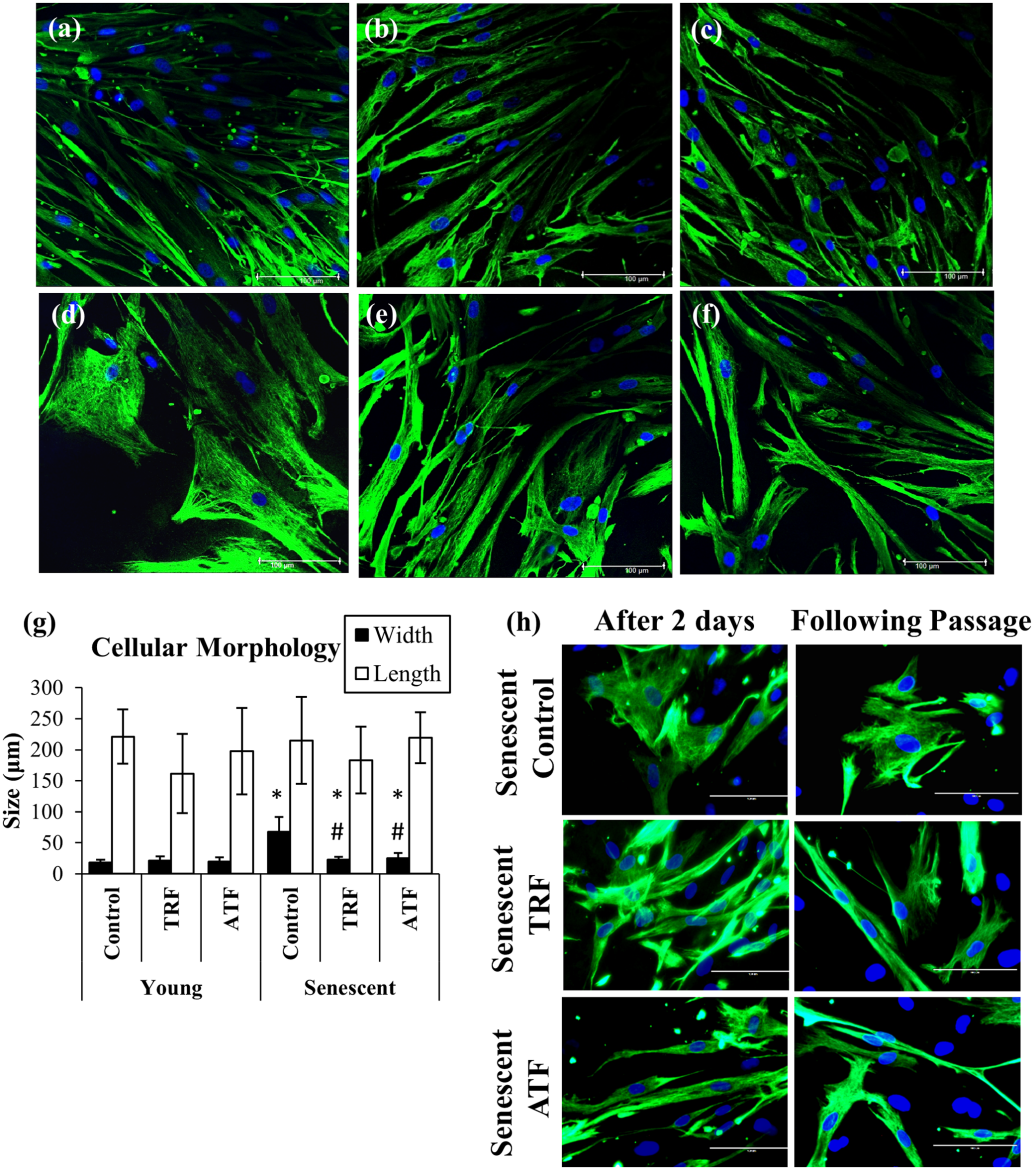
Effects of replicative senescence and vitamin E treatment on myoblasts phenotype. The photomicrographs of myoblasts were taken from a young control (a), TRF-treated young (b), ATF-treated young (c), senescent control (d), TRF-treated senescent (e) and ATF-treated senescent (f) cells (magnification: 400×). Myoblasts were stained for desmin (green) and Hoechst (blue). Both TRF- and ATF-treated senescent myoblasts resembled the morphology of young cells. The width and length of cells were measured (g). The width of senescent myoblasts significantly increased in the untreated control and decreased with TRF and ATF treatment. The spindle-shaped cells can be maintained in culture for two days after withdrawal of treatments and retained in the following passage (h). *p<0.05 significantly different compared to untreated young myoblasts, ^#^p<0.05 significantly different compared to untreated senescent myoblasts, with *post-hoc* Dunnett T3. The data are presented as the means ± SD, n = 30.

Myoblasts were spindle shaped when young but transformed into large and flat cells with a prominent intermediate filament network at the senescent stage (Fig 4a and 4d). Senescent cells exhibited a significantly higher ratio of cytoplasm to nucleus content than did young cells, as manifested by increased width during replicative senescence (p<0.05) (Fig 4g). However, both TRF- and ATF-treated senescent myoblasts retrieved the young-like morphology with the presence of more spindle-shaped cells (Fig 4e and 4f). Moreover, the average width of senescent myoblasts that were treated with both TRF and ATF significantly decreased compared to that of the untreated control (p<0.05) (Fig 4g). The spindle-shaped cells can be maintained in culture for two days after withdrawal of treatments and being observed in the following passage (Fig 4h).

### Reversal of Replicative Senescence by TRF and ATF

**Fig 5:**
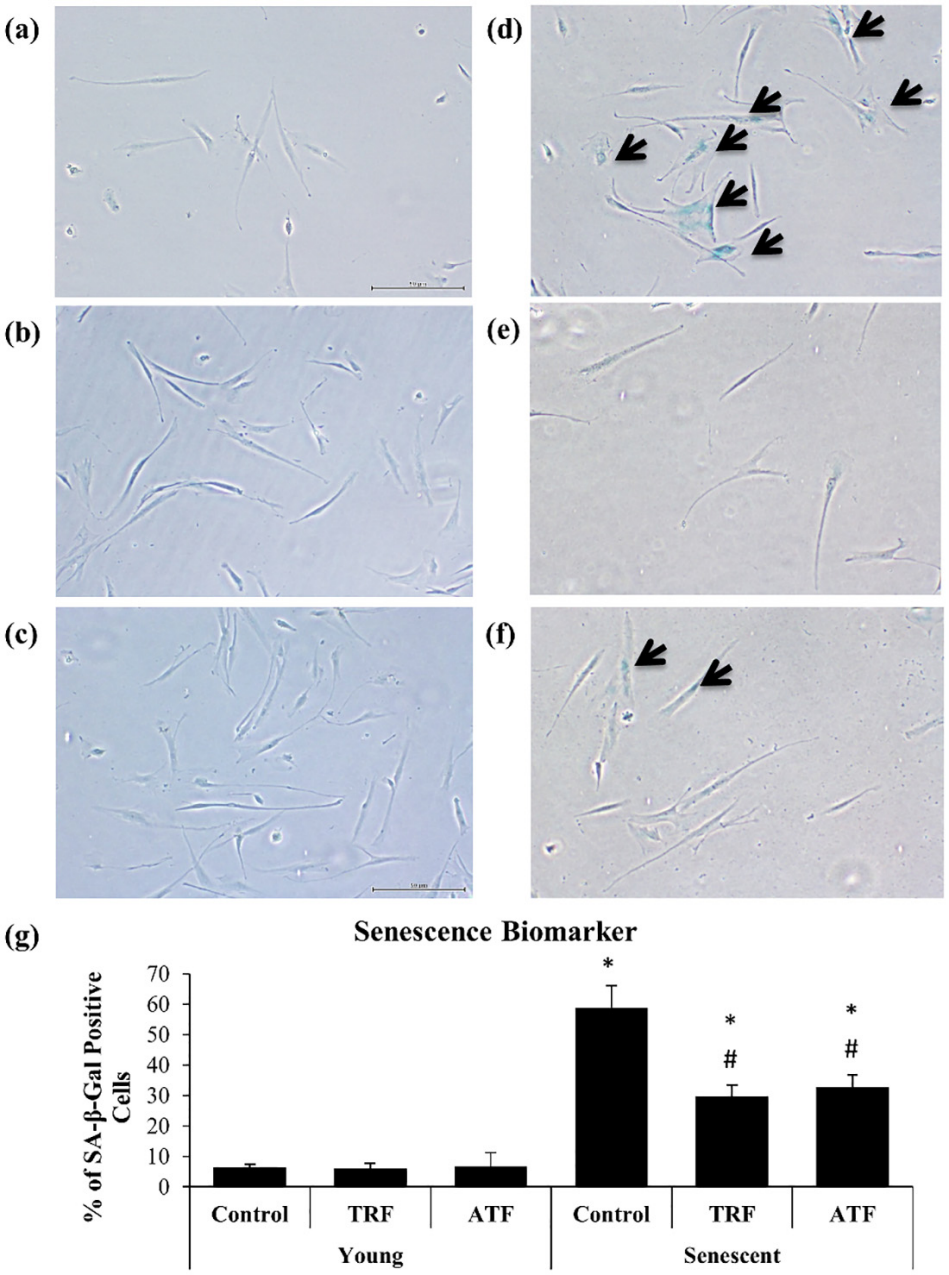
Effects of replicative senescence and vitamin E treatment on senescence biomarker. The photomicrographs of myoblasts were taken from young control (a), TRF-treated young (b), ATF-treated young (c), senescent control (d), TRF-treated senescent (e) and ATF-treated senescent (f) cells (magnification: 40×). Most of the senescent control myoblasts were stained positive for SA-β-gal (blue stained), as indicated by the arrow. The percentage of blue-stained cells was determined (g). TRF and ATF significantly reduced the number of blue-stained cells of senescent myoblasts. *p<0.05 significantly different compared to untreated young myoblasts, ^#^p<0.05 significantly different compared to untreated senescent myoblasts, with *post-hoc* Tukey HSD. The data are presented as the means ± SD, n = 3.

Senescent myoblasts were stained positive for SA-β-gal (Fig 5d–5f). The percentage of SA-β-gal-positive cells is shown in Fig 5g. The SA-β-gal-positive cells were markedly increased in senescent myoblasts (58.67% ± 7.5) compared to young cells (6.33% ± 1.2) (p<0.05). The percentage of SA-β-gal-positive cells significantly decreased to 29.67% ± 3.8 and 32.67% ± 4.0 (p<0.05) with TRF and ATF treatment compared to the untreated groups.

### Superior Effects of TRF in Promoting Cell Differentiation in Senescent Myoblasts

**Fig 6:**
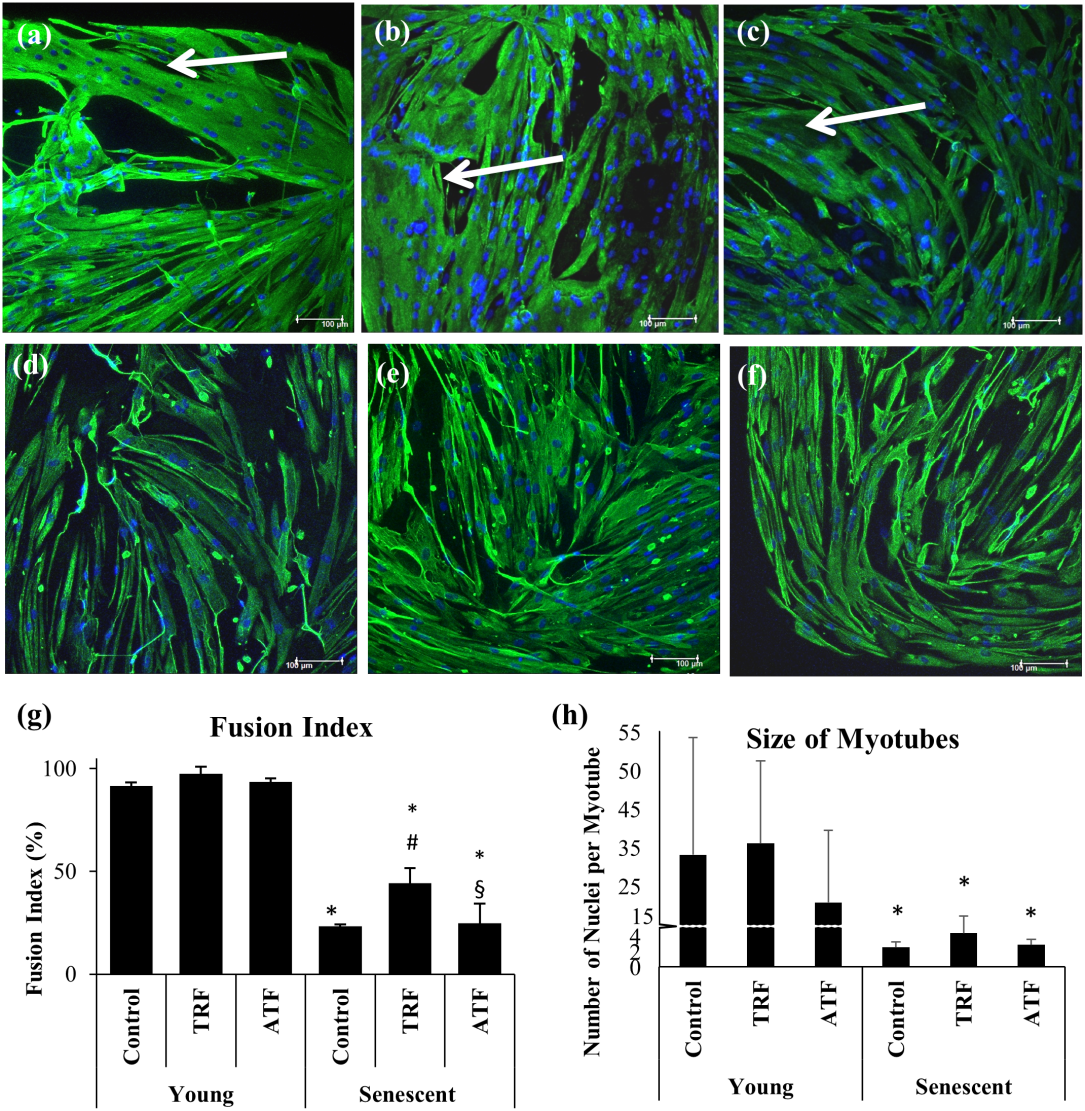
Effects of replicative senescence and vitamin E treatment on the differentiation capacity of myoblasts. The photomicrographs of myotubes were taken from young control (a), TRF-treated young (b), ATF-treated young (c), senescent control (d), TRF-treated senescent (e) and ATF-treated senescent (f) cells (magnification: 200×). Desmin was stained green, and the nuclei were stained blue (Hoechst). The myotubes that formed from young myoblasts were significantly bigger than the myotubes from senescent myoblasts. The fusion index (g) and the size of myotubes (h) were determined to evaluate the efficiency of muscle differentiation. TRF significantly increased the fusion index (n = 3), which was not shown with ATF treatment. No changes was observed in the size of the myotubes that formed (n = 12) with the TRF and ATF treatments. *p<0.05 significantly different compared to young control, ^#^p<0.05 significantly different compared to senescent control, ^§^p<0.05 significantly different compared to TRF-treated senescent myoblasts, with *post-hoc* Tukey HSD. The data are presented as the means ± SD.

Young and senescent myoblasts were allowed to differentiate for 9 days to form myotubes. Young myoblasts fused together, forming large and branched multinucleated myotubes (Fig 6a). Senescent cells, however, formed smaller myotubes with fewer branches compared to young cells (Fig 6d), indicating an inefficient differentiation process during the replicative senescence of myoblasts, which is similar to sarcopenic muscle.

A significant decrease in the fusion index and size of myotubes was observed in senescent myoblasts (Fig 6g and 6h). Approximately 91.66% of young myoblasts turned into myotubes, but only 23.17% of senescent myoblasts were able to fuse and form myotubes (Fig 6g). Large, branched multinucleated myotubes were formed from young myoblasts with approximately 25 nuclei per myotube, while much smaller myotubes were formed during replicative senescence with the presence of 2.5 nuclei per myotube (Fig 6h).

The multinucleated myotubes that formed from senescent myoblasts, however, were improved with TRF and ATF treatment (Fig 6e and 6f), even though they were still smaller in size and with fewer branches compared to young myoblasts. An analysis of the fusion index demonstrated that TRF treatment significantly promoted cell differentiation during cellular senescence, as indicated by a significantly increased fusion index in TRF-treated myoblasts (p<0.05) (Fig 6g). ATF, however, did not produce similar effects.

### Modulation of MRFs at the Early Phase of Myogenic Differentiation

**Fig 7:**
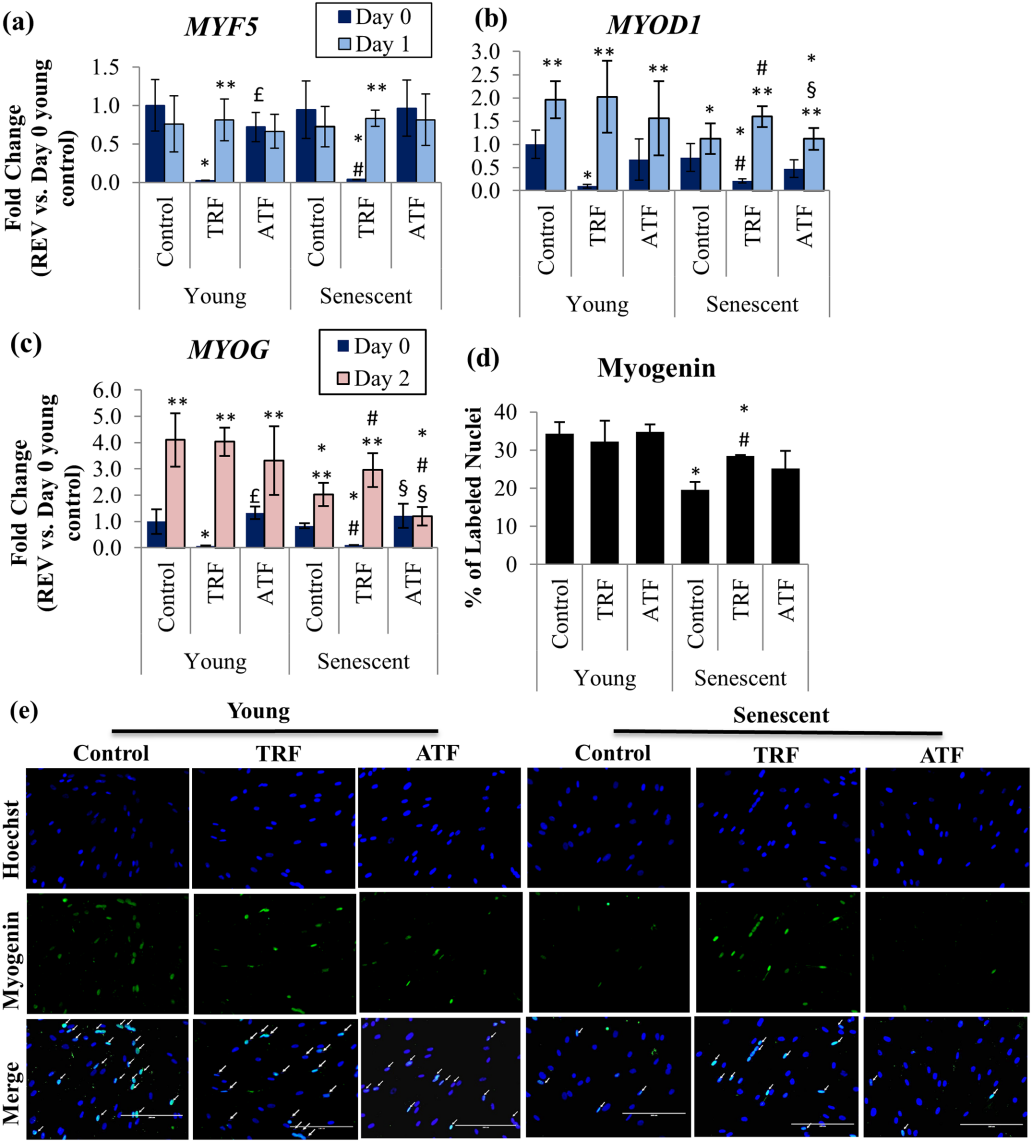
Effects of replicative senescence and vitamin E treatment at the early phase of myogenic differentiation. The *MYF5* (a) and *MYOD1* (b) mRNA expression levels on day 0 and day 1 of differentiation were determined, while the *MYOG* (c) mRNA expression level was determined on day 0 and day 2 of differentiation. The percentage of nuclei that stained for myogenin (green) on day 3 of differentiation is shown in (d). Photomicrographs were taken from all groups using a fluorescence microscope (magnification: 200×) (e). TRF significantly increased the number of myogenin-labeled nuclei on day 3 of differentiation, as indicated by arrows. *p<0.05 significantly different compared to young control at corresponding day of differentiation, ^#^p<0.05 significantly different compared to senescent control at corresponding day of differentiation, ^£^p<0.05 significantly different compared to TRF-treated young myoblasts at the corresponding day of differentiation, ^§^p<0.05 significantly different compared to TRF-treated senescent myoblasts at corresponding day of differentiation, **p<0.05 significantly different compared to the corresponding treatment at day 0 of differentiation. The data are presented as the means ± SD.

The modulation of MRFs expression by TRF and ATF during the replicative senescence of myoblasts was also investigated by determining the expression of *MYF5*, *MYOD1* and *MYOG* mRNA at the early phase of differentiation in young and senescent myoblasts (Fig 7a–7c). *MYOG* mRNA expression was upregulated at day 2 of differentiation induction in both untreated young and untreated senescent myoblasts (p<0.05), while *MYOD1* expression was upregulated at day 1 of differentiation in untreated young myoblasts only (Fig 7b and 7c). The expression of *MYOD1* and *MYOG* mRNA, however, was lower in untreated senescent myoblasts compared to untreated young cells (p<0.05).

Treatment with TRF down regulated *MYF5*, *MYOD1* and *MYOG* in young and senescent myoblasts at day 0 of differentiation induction compared to the untreated young control (p<0.05). At day 1 and day 2 of differentiation induction, the expression of *MYF5*, *MYOD1* and *MYOG* in both young and senescent myoblasts significantly increased (p<0.05) (Fig 7a–7c).

No significant changes were observed in the expression of *MYF5*, *MYOD1* and *MYOG* at day 0 of the differentiation induction of young myoblasts with ATF treatment compared to untreated control. After differentiation induction, ATF treatment caused upregulation to *MYOD1* and *MYOG* mRNA in young myoblasts. Only *MYOD1* was upregulated in senescent myoblasts at day 1 of differentiation induction (p<0.05) (Fig 7a–7c).

Myogenin protein expression significantly decreased in untreated senescent myoblasts compared to untreated young cells (p<0.05) (Fig 7d and 7e). TRF treatment significantly increased the expression of the myogenin protein in senescent myoblasts (p<0.05), while no significant changes were observed in the expression of myogenin with ATF treatment (Fig 7d and 7e).

### Modulation of MRFs during Myogenic Differentiation Phase

**Fig 8:**
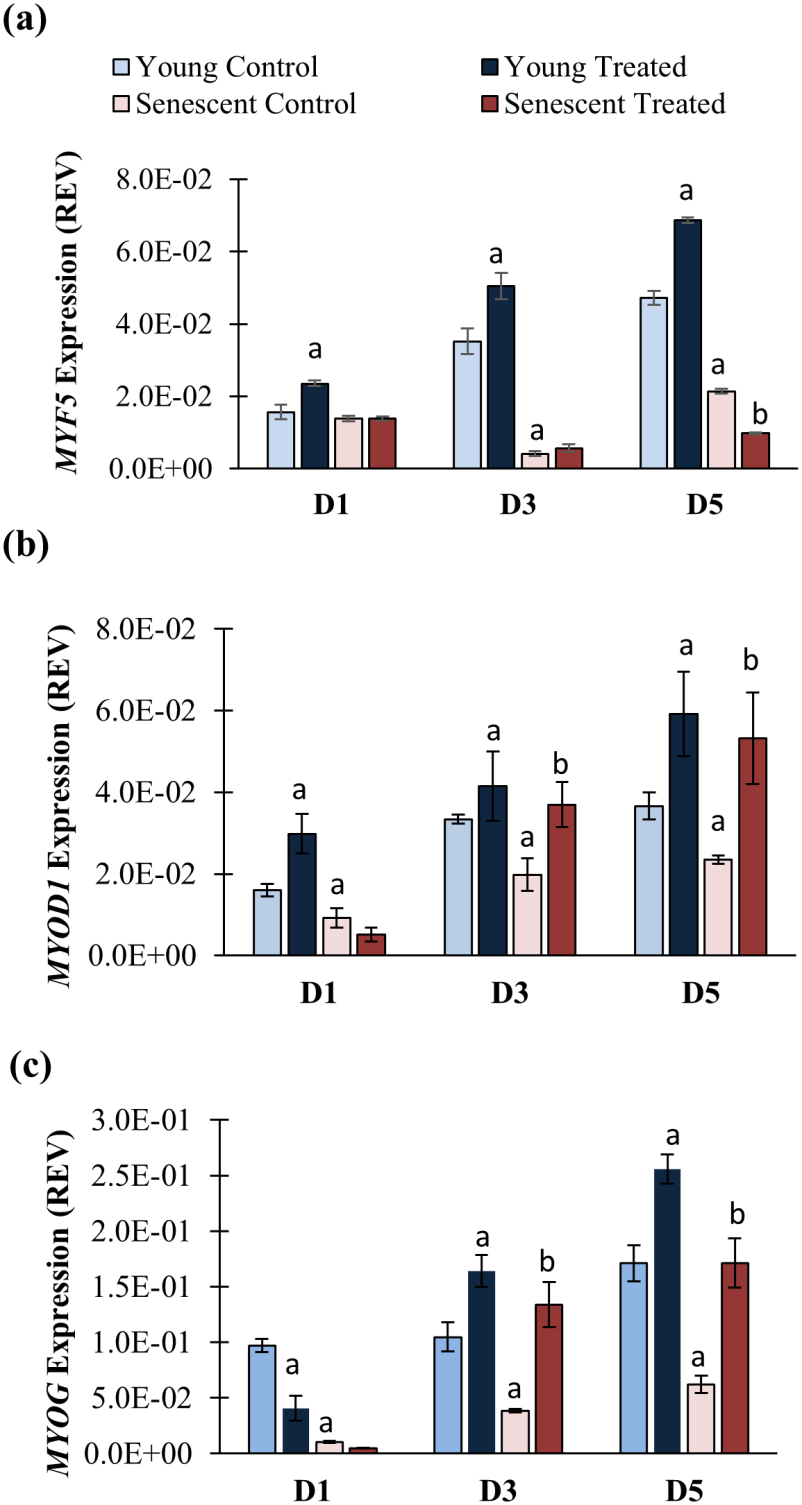
Effects of TRF on the MRFs mRNA expression levels during 5 days of differentiation induction. The *MYF5* (a), *MYOD1* (b) and *MYOG* (c) mRNA expression levels in senescent control were significantly lower than young control (p<0.05). TRF significantly increased the expression of both *MYOD1* and *MYOG* mRNA at day 3 (D3) and day 5 (D5) of differentiation, resembled the expression in young control, while the expression in senescent myoblasts remained low, even after 5 days of differentiation. ^a^p<0.05 significantly different compared to young control at corresponding day of differentiation, ^b^p<0.05 significantly different compared to senescent control at corresponding day of differentiation, The data are presented as the means ± SD.

To further validate the effect of TRF on MRFs during differentiation, expression of MRFs were determined in an extended time course and enhanced treatment protocol, in which myoblasts were treated with 50 μg/ml of TRF again during differentiation phase. Myoblasts from other donor (a 16-year-old male Caucasian) was used. The gene expression of MRFs (*MYF5*, *MYOD1* and *MYOG*) at day 1 (D1), day 3 (D3) and day 5 (D5) of differentiation induction were determined (Fig 8a–8c). The expression of *MYF5* mRNA was significantly decreased in senescent myoblasts at day 3 and day 5 of differentiation induction (p<0.05) (Fig 8a) while the expression of *MYOD1* and *MYOG* mRNA was significantly lower in senescent myoblasts at day 1 till day 5 of differentiation induction as compared to untreated young myoblasts (p<0.05) (Fig 8b and 8c).

The expression of MRFs was modulated with TRF treatment. In TRF-treated senescent myoblasts, both *MYOD1* and *MYOG* mRNA were significantly upregulated at day 3 and day 5 of differentiation induction as compared to untreated control (p<0.05) (Fig 8b and 8c). However, the expression of *MYF5* mRNA was significantly lower in TRF-treated senescent myoblast at day 5 of differentiation as compared to senescent control (p<0.05) (Fig 8a). TRF treatment also upregulated *MYF5* and *MYOD1* mRNA in young myoblasts at day 1 till day 5 of differentiation induction as compared to untreated control (p<0.05) (Fig 8a and 8b) while *MYOG* mRNA was upregulated at day 3 and day 5 of differentiation induction (p<0.05) (Fig 8c).

### Reduction of Free Radicals Generation by TRF

**Fig 9:**
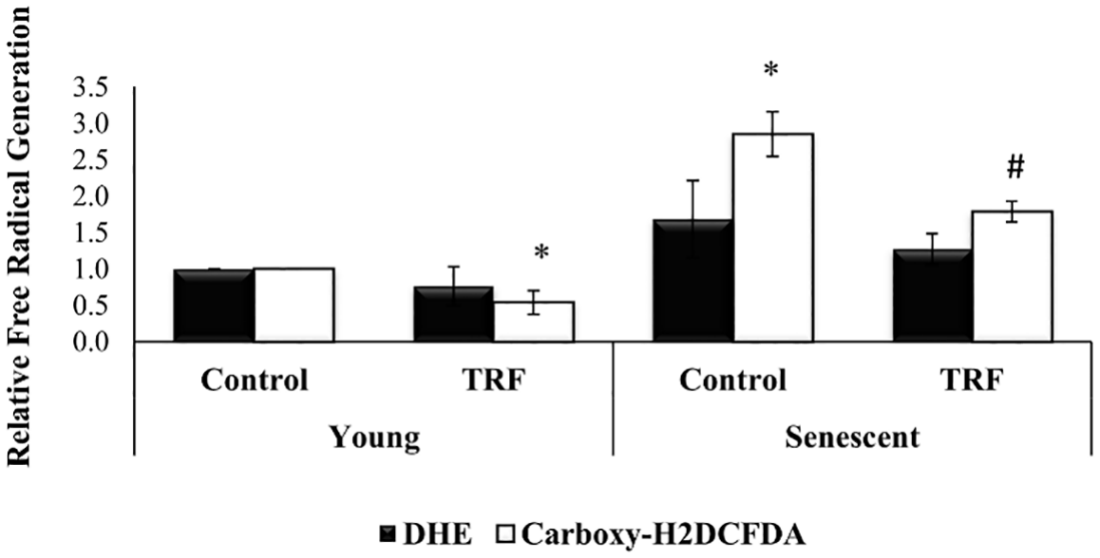
Effects of replicative senescence and TRF treatment on intracellular ROS generation. ROS was normally generated in myoblasts, however the elevated ROS level may disturb proliferation and survival of cells. The amount of intracellular ROS was significantly increased in senescent myoblasts. The fluorescence intensity of positive stained cells in senescent myoblasts was significantly reduced in TRF-treated cells, revealing free radical scavenging properties exerted in TRF. *p<0.05 significantly different compared to young control, ^#^p<0.05 significantly different compared to senescent control, with *post-hoc* LSD test. Data are presented as mean ± SD, n = 3.

To further elucidate the antioxidant effect of TRF, generation of free radicals or reactive oxygen species (ROS) was determined, in which the myoblasts were labelled with DHE (in orange) and carboxy-H_2_DCFDA (in green). The percentage of cells which were stained positive with carboxy-H_2_DCFDA was gradually increased from young to senescent. Quantitative analysis showed a significantly increased in carboxy-H_2_DCFDA-stained senescent myoblasts (2.85 fold) as compared to young myoblasts (p<0.05) (Fig 9). TRF treatment significantly reduced the amount of intracellular ROS generation in both young and senescent myoblasts, effectively (p<0.05) (Fig 9).

## Discussion

The tocotrienol-rich fraction (TRF) has been shown to have not only anti-aging properties in an *in vitro* study [23] but also beneficial effects against aging in animal models [24] and healthy older adults [25]. Recently, positive effects of TRF were highlighted in stress-induced premature senescent (SIPS) myoblasts following a 24-hour treatment [26], acquitting its free-radical-scavenging power. The mechanism by which TRF reduces senescence phenotypes in the muscle may not only involve combating oxidative stress, but is possibly associated with its regenerative capacity. In this study, we revealed novel insight into the potential of vitamin E for improving myoblasts proliferation and differentiation for the prevention of *in vitro* replicative senescence.

A slight modification of skeletal muscle homeostasis may lead to unsuccessful muscle renewal as in aging and pathological dystrophic muscle. In brief, myoblasts undergo the cellular life path starts with an exponential phase that then slows and reaches a finite proliferation state, indicating the replicative senescence phase [21,27]. However, caution is required when performing the serial passaging of myoblasts, as fibroblasts may swarm in and affect the myoblasts culture [28]. Thus, in conducting the experiment, myogenic purity must be maintained, even when cells reach replicative senescence.

The number of proliferating myoblasts is represented by the total BrdU incorporated into the cells, which gradually decreased with the increased population doubling over time. Interestingly, the amount of these active cells very much depends on the donor’s age [9,21]. Moreover, old-individual-derived myoblasts had a slower response to proliferative stimuli, which may contribute to the prolonged proliferation period in senescent cells [9]. In relation to the proliferative capacity, studies have shown that aged myoblasts in culture share the same characteristics with old individual-derived myoblasts, thus indicating the perception of regenerative capacity during aging, which can be modulated by intervention. Our data show that TRF not only increases cell viability but also enhances the proliferation capacity of senescent myoblasts. The total number of proliferating cells, however, did not increase with ATF treatment in senescent myoblasts, even though the same dosage of ATF retained more viable cells than did untreated control myoblasts. These findings indicate the potential of TRF in improving the proliferation capacity of senescent myoblasts in culture.

To further verify the capability of both vitamin E treatments in rescuing senescent myoblasts, the senescence biomarker SA-β-gal was used to identify the presence of senescent cells [22]. Our results show the association between total SA-β-gal-positive cells and total population doubling, indicating the accumulation of senescent cells with increased cell expansion as previous study [29]. Both TRF and ATF dramatically decrease SA-β-gal activity in senescent cells, signifying aging reversal effects on myoblasts in culture. These findings are supported by previous reports that showed a similar decrease in SA-β-gal expression with TRF treatment in human diploid fibroblasts [23] and H_2_O_2_-induced myoblasts [26].

Apart from a significant reduction in senescence biomarkers, the eradication of typical senescence morphology further demonstrated the anti-aging effects of TRF and ATF. Senescent myoblasts normally display an enlarged and flattened morphology with the presence of more intermediate filament networks [21,29]. Elevated extracellular matrix degradation and excessive protein loss have been reported to cause failure in ultra-structural preservation, consequently presenting the unique morphology of senescent cells [30]. However, senescent myoblasts that were treated with either TRF or ATF showed a cellular morphology that resembled that of young cells, whereby more spindle-shaped cells were present, suggesting improvement in cellular physiology by both vitamin E treatments.

There is a connection between the age of the donor and the differentiation process in myoblasts [14]. Such imperfection will decelerate muscle regeneration, as observed in aging. Similar effects were shown in myoblasts that were derived from Duchenne muscular dystrophy (DMD) patients [31] and patients with myotonic dystrophy type 2 [32]. These reports supported our findings, which displayed an incomplete differentiation process in senescent myoblasts [13,14], and may explain the molecular processes that lead to muscle atrophy in aging and muscular diseases.

Our data show that the differentiation defect in senescent myoblasts is partially revived by TRF treatment. A similar finding reported that the rejuvenation of aged satellite cells can be promoted by exposure to youthful niches [10]. In addition, myoblasts that were derived from old individuals failed to differentiate when exposed to autologous serum but differentiated into myotubes in the presence of young serum, indicating that aged myoblasts were saved by circulating factors that were present in young serum [9]. In our study, senescent myoblasts regained young features with TRF treatment, which may provide a permissive environment for optimum differentiation.

Replicative senescence deregulates the expression of myogenic-differentiation-related key transcription factors, resulting in impaired myogenic differentiation as shown by differentially expressed MRFs in young and senescent myoblasts [13]. During the early phase of differentiation, the expression of Myf5, MyoD and myogenin is delayed, and their expression levels at peak are still lower than in young myoblasts, signifying the alteration of appropriate signaling for differentiation in senescent cells [13]. Previous study reported a statistically upregulation of *MYOD* at day 1 and returned to baseline at day 2, along with *MYOG* mRNA expression significantly elevated from day 2 to day 6 in culture [33]. Thus, in our study, we observed a delay in *MYOD1* and *MYOG* mRNA expression at day 1 and day 2 respectively, as well as decreased myogenin protein expression in senescent myoblasts in the early differentiation state. Moreover, we showed that the MRFs mRNA expression remained lower in senescent myoblasts compared to young myoblasts up to 5 days of differentiation induction, which are similar to findings from a previous study [13]. In another study, a lesser induction of myogenin and MyoD in old-individual-derived muscle was observed, revealing a setback on MRFs expression during aging [34].

In our study, surprisingly, the basal *MYF5* mRNA expression levels in TRF-treated senescent myoblasts were noticeably low. Previously, the *MYF5* mRNA is found highly expressed in G0 myoblasts and decreased during the G0/G1 transition and persist during the rest of cell cycle progression [35]. Therefore, the expression of *MYF5* may due to the dynamic of cell cycle. Besides, satellite cells in skeletal muscle are heterogeneous stem cell pool that consist of different subpopulations of cells [6] that are regenerated from the asymmetric division of satellite cells [36]. Pax7^+^ satellite cells with Myf5^-^ expression can undergo asymmetric division and produce both Pax7^+^/Myf5^-^ and Pax7^+^/Myf5^+^ satellite cell populations, demonstrating the role of Pax7^+^/Myf5^-^ satellite cells in maintaining the muscle stem cell niche [36]. Thus, low level of *MYF5* mRNA expression may be explained by this situation, even though further studies may be required to elucidate the detailed mechanism involved. However, with differentiation induction, the decreased *MYF5* mRNA expression in TRF-treated senescent myoblasts returned to the normal level as in the young control. This finding agrees with a recent report that found Myf5 to be expressed in differentiating cells [37].

The results of our study also demonstrate that *MYOD1* and *MYOG* mRNA basal expression levels in senescent myoblasts were suppressed with TRF treatment. Subsequently, these mRNA expression levels increased in response to differentiation induction. Interestingly, comparison between wild type and *MYOD*^*-/-*^ satellite cells revealed that cell cycle progression was sustained in *MYOD*^*-/-*^ satellite cells [38]. On the other hand, *MYOG* knock-down (*MYOG*_kd_) upregulates genes that are involved in cell proliferation [39]. Thus, the suppression on both *MYOD1* and *MYOG* mRNA basal expression by TRF may favor myoblasts proliferation in the absence of differentiation stimuli.

Myogenin expression is chiefly dependent on MyoD [40]. Thus, the modulation of MyoD expression during replicative senescence as observed in this study would affect myogenin expression. Both MyoD and myogenin have a vital regulatory role in retaining myogenic differentiation [13,38,39]. Thus, a prompt increase in *MYOD1* and *MYOG* mRNA expression which was observed immediately after differentiation induction in TRF-treated senescent cells, may indicate that myoblasts cells were rescued from senescence and more inclined to differentiation in response to stimuli. The increased expression of the myogenin protein that was observed in this study further demonstrates that TRF improves muscle differentiation. In addition, both *MYOD1* and *MYOG* mRNA in TRF-treated senescent cells were elevated during the 5 days differentiation induction strengthening the fact that treatment with TRF during the early stage of differentiation promotes myogenic differentiation.

In this study, we attempted a nutritional approach to ameliorate senescence-associated aberration in myoblasts. To date, research findings have shown a link between vitamin E and muscle health; for instance, a population-based study and a chronic deprivation rodent model have reported that the adequate daily intake of vitamin E was correlated with muscle performance [19,20]. Sufficient vitamin E also aided in skeletal muscle survival, even in the presence of massive ROS. These findings could be attributed to the properties of vitamin E, which acts as a stabilizer for lipid membrane and scavenges ROS effectively [18]. Thus, vitamin E could be beneficial for ameliorating muscle degeneration in ageing or muscular diseases. In our study, accumulation of ROS was observed in senescent myoblasts which strengthened the fact that oxidative stress engendered age-related cell damage [41–43]. Our results demonstrated that TRF was able to reduce the accumulation of ROS in senescent myoblasts, revealing its potential in protecting myoblasts against oxidative stress during replicative senescence.

Previous studies have reported that tocotrienols have a different structure that makes it penetrate the membrane more easily than tocopherols and exerts a more potent antioxidant effect [44,45]. Compared to tocopherol, tocotrienols can efficiently be recycled and taken up by the cells [44,46]. These features may contribute to the superior effectiveness of tocotrienols in some circumstances, as has been shown in a study in which ATF required a higher dosage to produce similar effects compared to γ-tocotrienols to preserve cell viability from H_2_O_2_ insults [47]. In our study, we found that a broad mixture of TRF had better effects than did ATF alone. This result is comparable to findings from a previous study [24]. Because a discrepancy exists between isomers, TRF may be more effective than a single isomer of vitamin E.

In summary, our study highlighted the effects of vitamin E in ameliorating senescence-associated phenotypes and promoting differentiation in myoblasts during replicative senescence. We found that senescent myoblasts exhibited altered morphology and accumulated ROS with impaired proliferation and differentiation capabilities that were distinguishable from young myoblasts. Treatment with vitamin E (both TRF and ATF) was able to retrieve the young-like features in senescent myoblasts. TRF, however, exerted better effects than did ATF in promoting myoblasts proliferation, as indicated by BrdU incorporation. TRF possessed higher muscle-differentiation-promoting properties compared to ATF, as shown by the formation of myotubes and its modulation of MRFs expression. The antioxidant effect of TRF was also shown in this study. In conclusion, both the TRF and ATF have the potential to protect myoblasts from replicative senescence; however, a superior effect was shown by the TRF. The findings of this study provide the initial benefits of the TRF that may contribute to future clinical translation, in which TRF can be potentially applied to sarcopenic muscle or dystrophic muscle. Although the TRF improves senescent myoblasts by restoring their regenerative capacity, further studies are required to determine its effects *in vivo*, either in humans or in an animal model.
